# Multi-Watt Near-Infrared Phototherapy for the Treatment of Comorbid Depression: An Open-Label Single-Arm Study

**DOI:** 10.3389/fpsyt.2017.00187

**Published:** 2017-09-29

**Authors:** Theodore A. Henderson, Larry D. Morries

**Affiliations:** ^1^Neuro-Laser Foundation, Centennial, CO, United States; ^2^The Synaptic Space Inc., Centennial, CO, United States; ^3^Neuro-Luminance Inc., Centennial, CO, United States; ^4^Dr. Theodore Henderson, Inc., Centennial, CO, United States

**Keywords:** major depression, near-infrared, laser, photobiomodulation, near-infrared light therapy, suicidal ideation, antidepressant

## Abstract

**Background:**

The treatment of depression has been hampered by low efficacy of antidepressant medications and safety concerns with alternative modalities. Recent work demonstrated that multi-Watt transcranial near-infrared light therapy (NILT) can effectively treat traumatic brain injury (TBI). The current objective is to explore multi-Watt NILT efficacy in a proof-of-concept study as a treatment for depression.

**Methods:**

Thirty-nine sequential patients treated for TBI between March 2013 and May 2017 provided depression self-assessment data and/or were administered the Hamilton depression rating scale. Each completed the Quick Inventory of Depression Symptomatology-Self Report (QIDS) before and after treatment. Patients received multi-Watt NILT using near-infrared lasers (810/980 nm at 8–15 W) applied to forehead and temporal regions bilaterally for 9–12 min to each area. Pre- and posttreatment scores were analyzed by paired *t*-tests.

**Results:**

All met QIDS criteria for mild to severe depression and 69% had prior antidepressant trials. For 36 of the 39 patients, after 16.82 ± 6.26 treatments, QIDS scores indicated a robust response (decrease of QIDS total score by ≥50%). For 32 of 39 patients, posttreatment QIDS scores indicated a remission from depression (decrease of QIDS total score ≤5). Overall, the QIDS score fell from 14.10 ± 3.39 to 3.41 ± 3.30 SD (*p* = 6.29 × 10^−19^). With 12 or fewer treatments, QIDS score dropped from 14.83 ± 2.55 to 4.17 ± 3.93. Patients receiving ≥13 treatments showed a change in QIDS score from 13.67 ± 3.64 to 3.11 ± 3.14. Those (*N* = 15) who received the entire treatment course within ≤8 weeks (5.33 ± 1.72 weeks) showed a change in QIDS score from 13.86 ± 3.14 to 4.5 ± 3.94. Suicidal ideation resolved in all, but two patients. Patients remained in remission for up to 55 months after a single course of treatment.

**Conclusion:**

This is the first report of high-powered NILT showing efficacy for depression. Multi-Watt NILT showed far greater efficacy and persistent benefit compared to low-power (<1 Watt) infrared light treatments. Patients saw benefit often within four treatments and resolution of depressive symptoms occurred within 4 weeks for some. These data raise an intriguing possibility—that multi-Watt NILT may be a safe, effective, and rapid treatment for depression comorbid with TBI and possibly primary major depression disorder. A double-blind, placebo controlled trial is warranted to verify these proof-of-concept data.

## Introduction

Depression is arguably the leading cause of disability and lost productivity worldwide with an estimated 350 million people suffering from depression globally and many going untreated. Treatment efforts have focused on psychotherapy and pharmacological interventions; however, growing evidence indicates that current antidepressant medications fall far short of adequately treating depression. Overall, only 35% of patients with major depression disorder (MDD) respond to the first serotonergic antidepressant (1, 2). Studies examining the sequential use of antidepressants for unresponsive MDD also have yielded disappointing results. For example, the STAR-D trials examined antidepressant response in patients unresponsive to a first trial of serotonergic antidepressant and achieved only a 13% response rate after a sequence of four different antidepressants with multiple mechanisms of action (2, 3).

One limitation of the pharmaceutical treatment of depression has been the excessive focus on monoaminergic agents—those affecting serotonin, dopamine, and norepinephrine. Despite relatively poor response rates (1, 2) and significant (2) to profound (4–6) side effects and risks, monoaminergic agents have been the mainstay of psychiatry, even in the face of growing evidence that the monoaminergic pathways have limited impact of depression, leading Nassir Ghaemi to state,

Psychiatry… use(s) hundreds of made-up labels for professional purposes, without really getting at the reality of what is wrong with the patient…We have a huge amount of neurobiology research now to conclude that the 20th century neurotransmitter theories of psychopharmacology basically are false. (7)

More aggressive interventions, such as electroconvulsive therapy (8), TMS (9), vagal nerve stimulation (10), and deep brain stimulation (11) have shown promise, but represent an impractical and risk-laden solution for depression worldwide.

In 2016, one of the authors (TAH), along with colleagues from Massachusetts General, reviewed the current theories on the causes of depression in the context of the potential of a new treatment modality (12). Biological mechanisms which may contribute to depression include: oxidative stress, decreased metabolism, inflammation, and neurodegenerative processes. Evidence supports elevated oxidative stress in patients with depression, including elevated levels of superoxide dismutase, involved in removal of toxic oxidative radicals (13) and decreased levels of catalase, which protects cells from oxidative damage (14). Recent studies of levels of nucleotide triphosphates in patients with depression have shown decreased cellular energy availability (15) and a correlation was demonstrated between antidepressant treatment response and restoration of larger nucleotide triphosphate totals (15). Proinflammatory cytokines are elevated in those with depression (16) and the role of inflammatory pathways in altering neurotrophic support may underlie multiple neuropsychiatric disorders, including depression (17). Depression is associated with a loss of neurons, reduced synapse numbers, and dearborization of dendrites in the hippocampus and frontal cortices (18–21). Patients with MDD often exhibit significant atrophy of the hippocampus (22, 23). These structural rearrangements can be partially reversed with monoaminergic antidepressants (20, 24) and some have proposed that hippocampal neurogenesis is essential for antidepressant effects (25). Prolonged antidepressant benefits of certain antidepressant agents appear tied to synaptogenesis and synaptic potentiation resulting from upregulation of the neurotrophin, brain-derived neurotrophic factor (BDNF) (26), increased expression of BDNF receptors (trkB), and activation of the mechanistic target of rapamycin pathway (27). Notably, trkB receptor inhibition prevents prolonged antidepressant effects (28). These prolonged effects potentially may represent persistent neuroplastic and neuroregenerative actions of BDNF (26).

One non-invasive treatment in use for pain, inflammation, headache, stroke, and traumatic brain injury (TBI) is near-infrared (NIR) light, which has effects on oxidative stress, metabolism, inflammation, and neurotrophin levels (12, 29–32). While not fully understood, the mechanisms underlying the therapeutic benefits of NIR light appear to depend upon the absorption of NIR photons in the wavelength range of 600–1,200 nm by cytochrome c oxidase in the mitochondria (30, 32, 33). This appears to initiate modulation of reactive oxygen species and activation of nuclear factor kappa B (a redox sensitive transcription factor) (34), which lead to the replication of mitochondrial DNA and nuclear early-response genes. In addition, NIR light in a fluence range of 0.9–36 J/cm^2^ induces decreased expression of proapoptotic genes, increased expression of antiapoptotic genes, and increased expression of neurotrophic factors, such as nerve growth factor and BDNF (29, 30, 32). Subsequently, synaptogenesis, dendritic arborization, and neurogenesis are increased [for reviews see Chung et al. (30) and Henderson and Morries (35)].

The early studies of transcranial NIR light therapy (NILT) for TBI or depression has yielded some informative results. Naesser et al. (36) described two patients with TBI who were treated over 4–60 months with low-power (0.5 W) NIR using 870 nm light-emitting diodes (LEDs) at a surface fluence of 13.3 J/cm^2^. While these patients experienced cognitive improvement, the benefits were only transient and required continued daily treatments to maintain (36). A larger group of eleven subjects with TBI were studied more systematically with 20 min treatments 3 times/week for 6 weeks using 870 and 633 nm wavelength NIR at a power of 0.5 W (37). Eight of these subjects also experienced depression, ranging from mild to severe. The scores on the Beck Depression Inventory-II indicated that three of the eight subjects with depression showed some improvement in depression symptoms, shifting from severe to moderate depression in one case and from moderate to mild depression in two cases over the 8-week course of treatment (37). It is unclear whether these benefits were transient or persistent. Schiffer et al. (38) treated 10 patients with treatment-resistant depression [mean Hamilton depression rating scale (HAM-D) total score 23.9 ± 8.8 SD] with a single application of low-power NIR. Using an 810 nm LED applied for 4 min to two sites on the forehead at a fluence of 60 J/cm^2^, a significant reduction in HAM-D score was observed. This benefit was transient with return of symptoms after 4 weeks (38). Cassano et al. (39) have reported similar findings in four adults diagnosed with MDD (mean HAM-D score 19.8 ± 4.35 SD). Treatment consisted of applying low-power 808 nm NIR light to four sites on the forehead for 2 min at each site with a fluence of 84 J/cm^2^. Patients received a total of six treatments and HAM-D scores dropped to 13.0 ± 5.35. This benefit also appeared transient (39). In contrast, Morries et al. (32) treated ten patients with multi-Watt NILT (8–15 W) and found marked and persistent improvement in multiple symptoms associated with TBI, including reductions in sleep disturbance, depressive symptoms, and anxiety. They have reported functional neuroimaging changes in a separate patient with long-standing TBI who was treated with multi-Watt NILT (40). Taken together, this literature suggests that NIR-induced neuroplasticity may be beneficial for both TBI and depression (whether intrinsic or secondary to TBI).

While these prior studies suggest a role for NIR light as a therapeutic modality for depression, the *transient* nature of the benefit in some studies warrants clarification. The distinction appears to lie in the use of low power NIR light emitted by LED devices versus multi-Watt NIR light emitters. NIR light from LED devices is generally under 1 W in power at the source. Our prior laboratory studies have shown that NIR light from LED devices does not penetrate the thickness of human skin (35). In contrast, multi-Watt NIR energy does penetrate at least 3–4 cm into the mammalian brain (35) or cadaveric brain (41). Thus, it is likely that only more powerful multi-Watt NIR light is capable of penetrating into the human brain (35, 41, 42), delivering appropriate fluence at depth in the brain, and sufficiently stimulating BDNF and other factors to produce a lasting antidepressant effect.

Our clinical work has focused on treating patients with TBI using multi-Watt NILT (32, 43). MDD is the most frequent psychiatric comorbidity after TBI with prevalence rates of 14–77% (44). We and others have found that a large proportion of patients with mild-to-moderate TBI experience some of the symptoms of depression, including low mood, sleep disruption, suicidal ideation, and anhedonia. For example, Fann et al. (45, 46) have reported a high prevalence of depressive symptoms within 1 year after a TBI. They examined 559 patients within 1 year of a TBI using structured clinical interview and found a cumulative rate of 53% for MDD as determined by the Patient Health Questionnaire. By comparison, the 12-month prevalence rate of MDD in the general population is 6.7% (45). In our published sample of patients treated using NILT for TBI, 90% of the patients had depressive symptoms and 100% had anxiety symptoms. Depressive symptoms may be part of persistent postconcussive symptomatology or may represent an Adjustment Disorder with Depressed Mood; however, both Fann et al. (45, 46) and Mauri et al. (44) used standardized structured clinical interviews and standardized scales in their determination of criteria for MDD.

Herein, we describe a series of 39 patients treated for TBI, but who also manifested depressive symptoms and met criteria for moderate to severe depression by Diagnostic Statistical Manual, fourth Edition (DSM-IV) criteria. All, but three, of the patients demonstrated considerable to complete improvement in their depressive symptoms as measured clinically and by multiple depression rating scales in response to multi-Watt NILT.

## Materials and Methods

Sequential patients who were seen in our outpatient clinic between March 2013 and May 2017 for the treatment of TBI also completed depression questionnaires in an unblinded proof-of-concept retrospective clinical study. The Quick Inventory of Depression Symptomatology-Self Report (QIDS) (47, 48) was performed before and after a course of treatment. Scores were analyzed as paired *t*-tests (Microsoft Excel 2010) after testing for violation of normality assumption by boxplot comparison. A one-tailed *t*-test was used as there had been no clinical evidence for NILT worsening depression. An *a priori* power analysis was not performed, although the *N* to detect a 50% change in QIDS score (definition of treatment response) is less than 10 subjects. In addition, patients seen between August 2013 and May 2017 were separately evaluated by a Board-certified psychiatrist for DSM-IV criteria for MDD with at least moderate depression (HAM-D total score between 14 and 28). HAM-D was repeated after treatment as part of standard assessment. Data from the HAM-D were analyzed by the same methods as those from the QIDS. This study was carried out in accordance with the recommendations of Denver University and all subjects gave written informed consent in accordance with the Declaration of Helsinki. IRB approval was obtained from Denver University for retrospective study.

Patients received multi-Watt NILT using NIR lasers with wavelengths of 810 and 980 nm and a power range of 8–15 W as previously described (32). Briefly, Class IV lasers, either the LT1000 (LiteCure, Newark, DE, USA), a 10 W adjustable NIR laser emitter with wavelengths of 810/980 nm capable of delivering continuous or pulsed NIR light, the Diowave 810 (Diowave, Riviera Beach, FL, USA), an adjustable NIR emitter up to 15 W with a wavelength of 810 nm capable of delivering continuous or pulsed NIR energy, or the Aspen Laser (Denver, CO, USA), an adjustable NIR emitter up to 15 W with wavelengths of 810 and 980 nm capable of delivering continuous or pulsed NIR energy were utilized. The fluence delivered to the skin of patients ranged from 55 to 81 J/cm^2^. No other treatment modalities (medications, exercise regimen, supplements) were added, discontinued, or changed while receiving NILT. Infrared light was applied to the scalp overlying the forehead and temporal regions bilaterally. Application to each area lasted 9–12 min and the total time for each session was 30 min. The number of treatments was variable, depending upon individual patient improvement and ranged from 8 to 34 treatments. Temperature of the skin surface was monitored with a laser digital sensor. A continuous sweeping motion was utilized to minimize skin heating and cover a larger area. Symptoms were monitored clinically and with instruments described above.

## Results

A total of 39 patients were treated with NILT for TBI and provided depression scale responses before and after treatment (see Table 1). Twenty were female (51%) and the mean age was 40.5 ± 16.9 years. Of the 39 patients, 27 had a prior diagnosis of depression by an outside clinician. 69% had taken antidepressant medication prior to evaluation for treatment and 74% were taking antidepressants during the course of NILT treatment. Average duration of treatment with traditional antidepressants exceeded 72 months and all 39 patients continued to have symptoms of depression. No patients started or stopped a medication during the course of treatment. Of the 39 patients, 9 had been deemed “treatment-resistant” by an outside clinician. Based on the power densities of 55–81 J/cm^2^ delivered to the skin of patients in our clinic, our laboratory data (25) indicate that at the depth of 3 cm into the human brain, we would be delivering 0.8–2.4 J/cm^2^. This is exactly within the range which has been shown to activate growth factors, other genes, and neuroregenerative processes within animal models (19, 20). Patients experienced significant improvement in their symptoms of TBI (TBI-related results for a portion of these patients have been reported elsewhere) (32).

**Table 1:** Patient demographics with duration of TBI and/or depression shown in years; percentage of patients taking one or more prior antidepressant or mood stabilizer listed below; percentage of patients taking an antidepressant or mood stabilizer at the beginning of treatment; percentage of patients who were taking medication at the outset of treatment who were able to discontinue medication after the completion of treatment; and the percentage of total patients who had been labeled as “treatment resistant” by an outside clinician prior to coming for treatment.

Variable	Female (*N* = 20)	Male (*N* = 19)	Total (*N* = 39)
Age (years)	39.0 ± 16.1	42.1 ± 17.7	40.5 ± 16.9
Race (% Caucasian)	90	89	90
TBI (%)	100	100	100
Duration of TBI (years)	7.38 ± 5.14	13.3 ± 3.35	10.26 ± 11.32
Depression (%)	80	63	69
Duration of dep. (years)	6.08 ± 3.35	11.55 ± 13.28	8.74 ± 9.84
Prior antidep. (%)	80	58	69
Current antidep. (%)	85	58	74
Stopped meds (%)	47	55	48
Treatment rsst (%)	20	26	23

*Medications included fluoxetine, paroxetine, sertraline, citalopram, escitalopram, bupropion, venlafaxine, lamotrigine, valproate, lithium, quetiapine, aripiprazole, brexpiprazole, risperidone, and gabapentin*.

**Figure 1:**
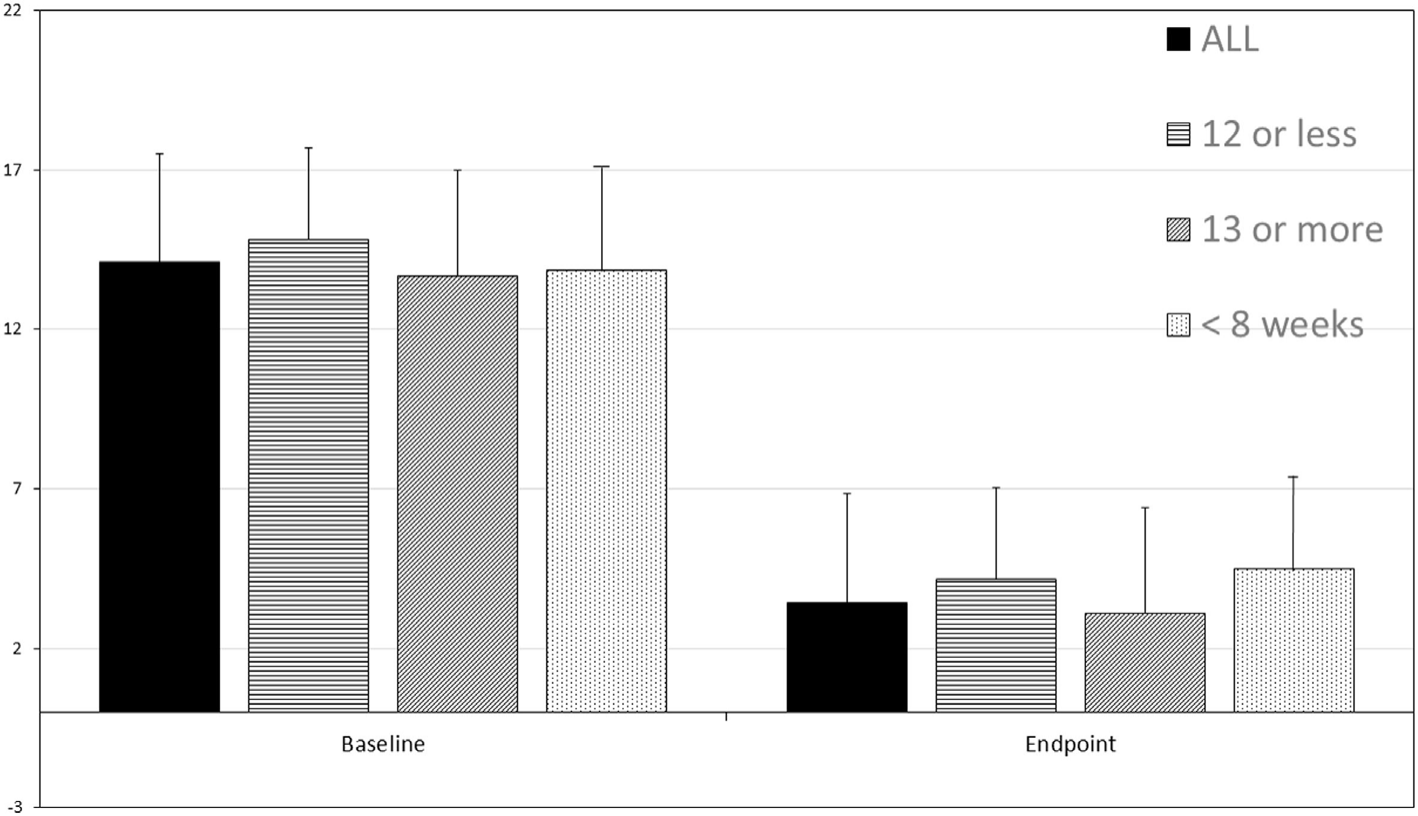
The Quick Inventory of Depression Symptomatology-Self Report (QIDS) score before and after transcranial multi-Watt near-infrared phototherapy treatment (Tx). Data for all 39 patients is shown, as well as data for those receiving 12 or fewer treatments (*N* = 12), ≥13 treatments (*N* = 27), and those treated in 8 weeks (*N* = 15). Mean and standard deviation are shown. *All posttreatment score decreases are statistically significant*. Total baseline QIDS score was 14.10 ± 3.39 and total endpoint QIDS total score 3.41 ± 3.30 SD (*p* = 6.29 × 10^−19^, paired *t*-test, one-tailed). Patients who received 12 or fewer treatments (*N* = 12) showed a change in QIDS score from 14.83 ± 2.55 to 4.16 ± 3.92 (*p* = 4.63 × 10^−6^). Patients who received ≥13 treatments (*N* = 27) similarly showed a change in QIDS score from 13.67 ± 3.64 to 3.11 ± 3.14 (*p* = 2.33 × 10^−13^). Patients who were treated in ≤8 weeks (*N* = 15) showed a robust response with a change in QIDS score from 13.87 ± 5.56 to 4.50 ± 3.94 (*p* = 5.56 × 10^−6^).

For 36 patients (92%), there was a dramatic decrease in depression symptoms. Patients often described benefit after the first four treatments. The mean number of treatments was 16.8 ± 6.26. For some patients, only eight treatments were given. All patients had QIDS scores in the range of mild to severe depression at baseline (see Figure 1). Overall, the baseline QIDS score was 14.10 ± 3.39, in the range of moderate depression. At endpoint, patients experienced a significant reduction of their depressive symptoms with a mean endpoint QIDS total score of 3.41 ± 3.30 SD (*p* = 6.29 × 10^−19^, one-tailed *t*-test, repeated measures). Thirty-six patients (92%) responded to the treatment (decrease of QIDS total score ≥ 50% from baseline) and 32 patients (82%) remitted from depression (QIDS total score ≤ 5). Patients who received 12 or fewer treatments (*N* = 12) showed a change in QIDS score from 14.83 ± 2.55 to 4.16 ± 3.92 (*p* = 4.63 × 10^−6^, one-tailed *t*-test, repeated measures). Patients who received ≥13 treatments (*N* = 27) similarly showed a change in QIDS score from 13.67 ± 3.64 to 3.11 ± 3.14 (*p* = 2.33 × 10^−13^, one-tailed *t*-test, repeated measures).

Of particular note, those who were treated in ≤8 weeks (*N* = 15) showed a robust response with a change in QIDS score from 13.87 ± 5.56 to 4.50 ± 3.94 (*p* = 5.56 × 10^−6^, one-tailed *t*-test, repeated measures). Indeed, six of the patients received the entire course of treatment (8–16 treatments, mean 10.63 ± 1.99) in ≤4 weeks. One patient categorized as a non-responder received the entire course of treatment in 4 weeks.

Suicidal ideation was present in 77% of the patients at baseline. Responses to QIDS question #12 (“Thoughts of death or suicide”) was 1.0 ± 0.79, with eight patients responding with a 2 (thinking of suicide “several times a week for several minutes”) or a 3 (thinking of suicide or death “several times a day in depth”). After treatment, two patients (non-responders) continued to endorse suicidal ideation. The mean response for question #12 of the QIDS posttreatment was 0.05 ± 0.22.

Ninety-two percent of patients endorsed feeling sad at baseline. The mean baseline response to QIDS question #5 pertaining to feelings of sadness was 1.87 ± 0.86. After treatment, the mean response to QIDS question #5 was 0.37 ± 0.63. Four patients did not achieve remission (QIDS scores ≥5) due to complaints of low energy, excessive sleep, or poor concentration, which can be residual symptoms of TBI. Three additional patients did not show treatment response. One patient who did not show treatment response went on to receive ketamine infusion therapy and experienced improvement in depressive symptoms. A second non-responder was lost to follow-up. The third non-responding patient had received ten treatments with NILT and then went on to receive ketamine infusion therapy, but after two infusions discontinued that treatment modality. This patient remained depressed and went on to receive a poly-pharmacy regimen, but remained depressed.

The effects of multi-Watt NILT on depression did not appear to be transient. We have follow-up interviews with 31 responder patients at posttreatment intervals of 2, 6, 12, and (in five cases) 55 months. One of the patients reported a return of depressive symptoms, although remaining free of TBI-related symptoms. At follow-up, none of the patients endorsed suicidal ideation when re-questioned using QIDS question #12. At follow-up, 62% reported not feeling sad (1 on QIDS question #5). The remainder endorsed intermittent feelings of sadness (2 on QIDS question #5).

Hamilton Depression Scale data reflected similar findings. The mean HAM-D score at baseline was 21.48 + 5.24 which is in the severely depressed range. After treatment, the mean HAM-D score was 6.0 ± 5.12 (*p* = 6.45 × 10^−13^, one-tailed *t*-test, repeated measures).

Adverse events were few. Six patients (15%) described headache after the initial one to three NILT treatments, but not with additional NILT treatments, thereafter. Eleven (28%) described feeling tired or fatigued after the initial one to three NILT treatments, but this resolved with further NILT treatment. Localized skin warming was noted by all patients, but was described as “comfortable.” Skin temperature increased no more than 3°C with rapid cooling after removal of the NIR light.

## Discussion

This is the first report of multi-Watt or high-powered infrared light therapy showing efficacy for depression symptoms. Of 39 patients, 36 experienced a treatment response. Overall, 92% responded to the multi-Watt NILT and 82% remitted from their depressive symptoms.

Patients did not experience discomfort or adverse reactions to the treatment. Localized skin warming was present, but benign. Headache and fatigue after the first few treatments were the only adverse events reported, but these side effects appeared to resolve with further treatment.

Time to response and time to remission were notable. Patients saw benefit often within four treatments. In two cases (one with moderate depression and one with severe depression), resolution of depressive symptoms was achieved with only eight treatments. Patients who received ≤8 weeks of treatment (*N* = 15) showed no significant difference from the entire group. Notably, these patients had a drop in QIDS score indicative of remission (13.87 ± 3.14 to 4.50 ± 3.94) within 8 weeks. Six patients (15%) were treated over the course of ≤4 weeks. Five of the six patients achieved remission of depressive symptoms. The remaining patient was a non-responder. The time to response (with exceptions already noted) was often more rapid than that found for typical antidepressants, usually 6–8 weeks. The rapid response is not dissimilar to what has been described for ketamine infusion therapy (26, 49, 50). The rapidity of response will need to be clarified in future studies.

Our 82% treatment remission rate is superior to that described for oral antidepressants (1, 2) and to that described by Schiffer et al. (60%) in 10 patients (38) and Cassano et al. (50%) in four patients (39) using low power infrared light. In addition, our posttreatment interviews for up to 55 months posttreatment show a large proportion of the patients remained free of depression symptoms. Therefore, these benefits do not appear to be transient, as was observed in studies of low-power NIR phototherapy (38, 39).

The magnitude, rapidity, and persistence of the response to the antidepressant effects of the multi-Watt (10–13 W) NILT may be explained by the superior penetration of multi-Watt NILT (25) compared to LED-based low-power (0.5 W) used in prior studies (38, 39). Our recent tissue studies demonstrate no penetration of low-level (0.5–6.0 W) NIR energy through 3 cm of skin, skull and brain (35). However, at 10–15 W, 0.45 to 2.9% of 810 nm light penetrated 3 cm of tissue. A 15 W 810 nm NIR device (continuous or non-pulsed) delivered 2.9% of the surface power density to 3 cm of depth. Pulsing at 10 Hz reduced the dose of light delivered to the surface by 50%, but 2.4% of the surface energy reached the depth of 3 cm (35). Based on the power densities of 55–81 J/cm^2^ delivered to the skin of patients in our clinic, our laboratory data indicate that at the depth of 3 cm into the human brain, we would be delivering 0.8–2.4 J/cm^2^. This range corresponds with fluence shown to activate growth factors, such as BDNF, and neuroregenerative processes in animal models (29, 30). These laboratory data support that not only is low-level NIR energy unlikely to penetrate significantly into the human brain, but that multi-Watt NILT, as used in the present study, is capable of delivering effective doses of NIR energy to at least 3 cm into the brain through skin, skull, and dura.

There are certain limitations to this study. It is an open-label series of patients receiving treatment as usual in our clinic. All of the patients were being treated for TBI with secondary depression, so it is unclear whether multi-Watt NILT would work equally well in patients with primary MDD without comorbidity. The symptoms responsive in this patient series might be equally well-explained as an Adjustment Disorder with Depressed Mood related to troublesome symptoms of TBI. Alternatively, the symptoms of depression may be too entangled in the postconcussive syndrome to be considered as a separate phenomenon from the overarching TBI-related syndrome. Lastly, it is an open-label study susceptible to placebo effect.

These open-label single-arm data raise the possibility that NILT may be a safe, effective, and rapid treatment for depression comorbid to TBI. Indeed, some of our patients responded within 4 weeks which is much more rapid than the response typical of standard oral antidepressants. Given the positive, but transient, antidepressant effects of low power infrared light (38, 39), these data suggest that multi-Watt NILT also may yield clinical benefit in primary major depressive disorder. At a time when Medicine is faced with a growing disappointment in traditional antidepressants (6, 7, 45) and alternative treatments such as TMS (8, 9), vagal nerve stimulation (10), and deep brain stimulation (51), which are expensive and difficult to obtain in the current insurance climate, offer only a marginal improvement in response rate. For example, TMS has a response rate of 48.7% and a remission rate of 33.6% in a recent meta-analysis (8). Deep brain stimulation, after the initial excitement, has demonstrated a response rate of 39.9% and a remission rate of 26.3% (51). Ketamine infusion therapy (26, 49, 50, 52) has shown early promise, but also considerable concerns and limitations. As an alternative, NILT appears to be a potentially safe and viable solution. These data are only a prelude. The patients were primarily in treatment for TBI; hence, the depressive symptoms may have reflected the impact of brain injury. A double-blind, placebo controlled trial is warranted to verify these preliminary data. Nevertheless, we have followed our responders (*N* = 36) for 31–55 months and they have remained depression-free. Given the depression relapse rate of 45% in untreated patients (1, 2, 4–6), this is an additional unique feature of multi-Watt NILT as a potential treatment modality for depression.

## Ethics Statement

This retrospective study of clinical data collected in the course of treatment as usual was carried out with written informed consent from all subjects for retrospective study. IRB approval was obtained from Denver University for retrospective study.

## Author Contributions

Authors participated fully in the treatment of patients, data collection, analysis of data, and preparation of manuscript.

## Conflict of Interest Statement

The authors declare that the research was conducted in the absence of any commercial or financial relationships that could be construed as a potential conflict of interest.
